# Cord blood-based neonatal genetic screening for thalassemia in Guiyang, China (2015): a cross-sectional study

**DOI:** 10.3389/fgene.2026.1883811

**Published:** 2026-09-24

**Authors:** Xiaoling Liang, Wen Xia, Siqi Long, Lei Hu, Caili Wang, Jingrong Zhang, Lei Zhang, Shu Mo, Danna Wei

**Affiliations:** 1 Department of Pediatric Hematology, Guiyang Maternal and Child Health Care Hospital, Guiyang Children’s Hospital, Guiyang, China; 2 Shenzhen Tsure Biotechnology Co Ltd., Shenzhen, Guangdong, China; 3 Department of Hematology and Oncology, The University of Hong Kong-Shenzhen Hospital, Shenzhen, China

**Keywords:** Thalassemia, neonatal screening, umbilical cord blood, carrier frequency, ethnic disparities

## Abstract

**Background:**

This study aims to assess the carrier frequency and molecular spectrum of thalassemia in neonates from Guiyang, China, a multi-ethnic region with historically limited population-level screening data.

**Methods:**

This single-center cross-sectional study recruited 1,000 consecutive umbilical cord blood samples from full-term neonates delivered at a tertiary maternal and child health hospital in Guiyang from January 2015 to December 2015. Genetic screening for thalassemia was conducted using commercial gene detection kits. Epidemiological data, including sex, age, ethnicity, place of origin, and routine blood test results, were obtained from the parents of all subjects. High-throughput sequencing was employed to re-examine cord blood samples from neonates whose parents had reduced MCV or a family history of thalassemia.

**Results:**

Of the 1,000 neonates, 74 were identified as thalassemia carriers, yielding an overall carrier rate of 7.4%. Specifically, 55 cases (5.5%) carried α-thalassemia genes, 18 cases (1.8%) carried β-thalassemia genes, and 1 case (0.1%) was a compound αβ-thalassemia carrier. Among α-thalassemia carriers, the three most frequent deletion genotypes were -α^3.7^ (45.45%), --SEA (27.27%), and -α^4.2^ (16.36%); non-deletional mutations included α-CS and α-WS. For β-thalassemia carriers, the most common mutations were CD17 (A→T) (44.44%), CD41-42 (-TTCT) (27.78%), and IVS-II-654 (C→T) (16.67%), with one rare domestic mutation (+40–43 [-AAAC]). Of note, 34 carriers were from ethnic minority groups, representing 10 distinct ethnicities. Carrier rates varied significantly by ethnicity: Han 6.28% (43/685), Buyi 17.33% (13/75), Miao 7.79% (6/77), Dong 26.32% (5/19), and Tujia 15.63% (5/32). Intergroup comparisons revealed significantly higher carrier rates among the Buyi, Dong, and Tujia populations compared to the Han population (all P < 0.05, χ^2^ test).

**Conclusion:**

This neonatal cord blood screening study in Guiyang reveals an overall thalassemia carrier rate of 7.4%, with α-thalassemia predominating. The -α^3.7^ deletion and CD17 (A→T) mutation were identified as the most frequent α- and β-thalassemia alleles, respectively. Carrier rates were higher in the Buyi, Dong, and Tujia populations compared to the Han population. Further epidemiological studies are needed to confirm the high prevalence of thalassemia in these ethnic groups.

## 1 Introduction

Thalassemia is a group of hereditary hemolytic anemias caused by defects in globin genes. Its pathogenesis is characterized by decreased or absent synthesis of globin chains, resulting in abnormal erythrocyte morphology, shortened red blood cell lifespan, hemolytic anemia, and various related complications (Khandros and Kwiatkowski, 2019; Musallam et al., 2024; Su et al., 2025). The disease exhibits distinct geographical distribution patterns, with global prevalence around the Mediterranean coast, Southeast Asia, and southern China (Modell and Darlison, 2008). In China, high-prevalence regions include Guangdong, Guangxi, Sichuan, Yunnan, and Guizhou, with carrier rates exceeding 10% in certain areas (Musallam et al., 2023; Wang et al., 2023). Clinically, thalassemia is categorized into mild, intermediate, and severe forms based on disease severity. Mild carriers are typically asymptomatic or exhibit only mild anemia, while severe cases often lead to death in infancy or childhood due to chronic hemolysis, recurrent infections, and organ dysfunction, imposing significant psychological and economic burdens on families and severely impacting regional public health (Baird et al., 2022). At present, treatment options for severe thalassemia remain limited. Long-term blood transfusions can sustain life, and allogeneic hematopoietic stem cell transplantation is the only curative treatment. The absence of other safe, radical therapies highlights the critical importance of early prevention (Shenoy and Thompson, 2016; Cappellini and Motta, 2017).

Given thalassemia’s hereditary nature, the core of its prevention and control lies in early screening, accurate diagnosis, and preconception and prenatal intervention. Establishing regional genetic background data is fundamental to prevention efforts. In recent years, provinces with high thalassemia prevalence in China have implemented population-wide screening programs to reduce the incidence of severe thalassemia births. Large-scale screenings in Guangdong, Guangxi, and Hunan have clarified local carrier rates and genetic distributions, providing a basis for targeted prevention strategies (Ying et al., 2022; Jiwu et al., 2023; Dan et al., 2024). However, Guizhou Province still has obvious deficiencies in thalassemia prevention and control. Prior to 2021, only limited population-level screening data were available, with the earliest epidemiological investigations confined to small-scale surveys of specific ethnic groups carried out in the mid-2000s using traditional hematological methods, which could only tentatively identify anemia types without precise genetic genotyping. As a result, comprehensive genetic characterization of the provincial population has remained unavailable for a prolonged period, hindering the formulation of evidence-based prevention strategies. Furthermore, the past 3 decades of frequent population migration and genetic evolution have made these fragmented historical data unable to accurately reflect the current carrier status. Although subsequent studies—including a 2021 survey of 18,309 neonates (carrier rate 12.90%) and a 2024 investigation among children <18 years old (overall carrier rate 25.0%)—have progressively filled some data gaps, they either focused on specific age groups or lacked the systemic coverage required for province-wide policy making (Mei et al., 2021; Yan et al., 2024). These persistent gaps underscore the pressing need for updated, large-scale, population-representative data to support modern prevention, diagnosis and intervention strategies.

The present single-center cross-sectional study based on 1,000 neonatal umbilical cord blood samples from Guiyang aims to calculate the overall thalassemia carrier rate among local newborns, systematically characterize the full molecular spectrum of α- and β-thalassemia pathogenic variants in multi-ethnic neonates, compare the differences in thalassemia carrier frequencies across different ethnic groups in Guizhou, and assess the diagnostic efficiency of the two-tier screening strategy combining conventional gene kit and NGS, so as to provide real-world epidemiological evidence for optimizing regional neonatal thalassemia prevention and genetic counseling programs.

## 2 Materials and methods

### 2.1 Study setting

This single-center research was carried out at a tertiary maternal and child health hospital in Guiyang, Guizhou. As the hospital with the highest local delivery and thalassemia screening throughput, its neonatal cohort can represent urban Guiyang newborns. The hospital’s certified molecular diagnostic laboratory is equipped with standardized cold-chain specimen management and has routine capacity for hemoglobinopathy testing.

### 2.2 Study design and period

This study adopted a single-center cross-sectional epidemiological screening design. All 1,000 umbilical cord blood specimens were consecutively recruited from newborns delivered at Guiyang Maternal and Child Healthcare Hospital between January 2015 and December 2015.

### 2.3 Study population and sampling

All eligible neonates were enrolled via random sampling throughout the study period. Sample size was statistically calculated based on cross-sectional epidemiological methodology. Since no local baseline neonatal thalassemia carrier rate data of Guiyang had been published at the time of this study, We adopted the reported β-thalassemia carrier rate of 5.40% (Yu et al., 2010) as the conservative baseline. Using a standard cross-sectional epidemiological formula with a 95% confidence level and an allowable absolute error of 3%, the calculated minimum sample size was approximately 218 neonates. Considering multi-ethnic subgroup analysis, potential sample loss and stable rare mutation frequency statistics, we expanded the target sample to 1,000 neonates to guarantee reliable inter-ethnic comparison and molecular spectrum analysis.

### 2.4 Laboratory investigations

Umbilical cord blood was collected from newborns immediately after delivery for genomic DNA extraction. Specimens were transferred to the molecular laboratory under standardized cold-chain packaging at controlled temperatures within a fixed transport timeframe. Residual purified DNA was preserved under defined short-term and long-term cryogenic storage conditions.

Genomic DNA was extracted from neonatal umbilical cord blood specimens using the QIAamp DNA Blood Mini Kit (QIAGEN, Hilden, Germany). Screening of thalassemia gene mutations was carried out by Yanneng Biotechnology-Shenzhen through PCR-RDB routine protocol that has been described in previous study (Yu et al., 2010). It covers 23 common Chinese thalassemia genotypes: 3 deletional α-thalassemia (-α^3.7^, -α^4.2^, and--SEA), 3 non-deletional α-thalassemia (Hb CS, Hb QS, and Hb WS), and 17 common β-thalassemia mutations.

Next-generation sequencing (NGS) was performed on cord blood samples from neonates whose parents exhibited reduced mean corpuscular volume (MCV) (<80 fL) to identify potential novel mutations. Targeted NGS was performed by BGI-Shenzhen using a validated custom globin gene panel. The panel covered full-length *HBA1*, *HBA2*, and *HBB* genes, additional globin loci, and key regulatory regions of α- and β-globin clusters, enabling comprehensive detection of pathogenic SNVs and indels. Library construction was conducted per standard protocols, and sequencing was carried out on the MGISEQ-2000 platform with paired-end 100 bp reads. The average target sequencing depth was maintained at ≥500× for core globin genes to ensure accurate variant calling. A total of 95 cord blood samples from neonates with parental low MCV indicators were subjected to supplementary NGS screening.

### 2.5 Statistical analysis

Raw data collation was performed using GraphPad Prism 10.0 (GraphPad Software, San Diego, CA, USA). Statistical analyses were performed using SPSS software (version 23.0). Descriptive data were expressed as mean ± standard deviation (SD). A two-tailed P-value <0.05 was considered statistically significant. The Shapiro-Wilk test was used to assess the normality of quantitative variables. If data were normally distributed (P > 0.05), Tukey’s HSD test was used for pairwise comparisons.

### 2.6 Ethical considerations

This study was approved by the Institutional Review Board of Guiyang Maternal and Child Healthcare Hospital and Guiyang Children’s Hospital (2022-67).

## 3 Results

### 3.1 Carrier frequency of α- and β-thalassemia in neonatal cord blood

A total of 1,000 umbilical cord blood samples from consecutive neonates were successfully genotyped for common α- and β-thalassemia mutations. Of these, 74 cases were identified as thalassemia carriers, resulting in an overall carrier rate of 7.4%. Stratification by thalassemia type revealed that α-thalassemia was the predominant form, with 55 carriers identified. β-Thalassemia carriers comprised 18 cases, while one neonate was found to carry both α- and β-thalassemia mutations (compound αβ-thalassemia) (Table 1).

**TABLE 1:** Overall thalassemia carrier status of newborns, Guiyang, China, 2015 (N = 1,000).

Genotype category	Number of cases (n)	Carrier rate (%)
α-thalassemia	55	5.5
β-thalassemia	18	1.8
Compound αβ-thalassemia	1	0.1
Total thalassemia	74	7.4

The total number of newborns screened was 1,000. Carrier rate is calculated as (number of cases/1,000) × 100%.

### 3.2 Spectrum and frequency of β-thalassemia mutations

Among the 18 β-thalassemia carriers, five distinct β-globin gene mutations were detected. The mutational spectrum was predominantly dominated by three alleles, accounting for 88.9% (16/18) of all β-thalassemia chromosomes. The most common mutation was CD17 (A→T), observed in 8 carriers (44.44% of β-mutated alleles), followed by CD41-42 (-TTCT) in 5 carriers (27.78%) and IVS-II-654 (C→T) in 3 carriers (16.67%). Notably, a rare domestic mutation, +40–43 (-AAAC), was found in one carrier (5.56%), representing a novel or previously unreported variant in this population. One remaining carrier (5.56%) harbored another rare or undetermined mutation not fully characterized in the current panel (Figure 1).

**FIGURE 1:**
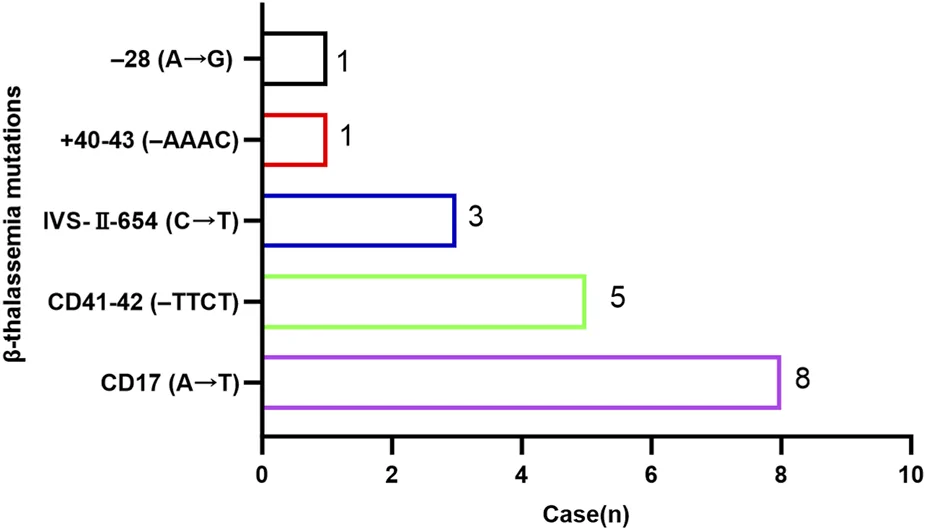
Distribution of β-thalassemia genotypes, Guiyang, China, 2015 (N = 18).

### 3.3 Deletion and non-deletion α-thalassemia genotypes in neonatal carriers

Among the 55 α-thalassemia carriers, five distinct α-globin genotypes were detected, consisting of three deletional and two non-deletional mutations. The deletional alleles predominated, accounting for the majority of α-thalassemia chromosomes. The most frequent deletion was -α^3.7^ (the 3.7 kb deletion), observed in 45.45% (25/55) of α-carriers, followed by--SEA (Southeast Asian deletion) in 27.27% (15/55) and -α^4.2^ (4.2 kb deletion) in 16.36% (9/55). Together, these three deletional genotypes represented 89.08% (49/55) of all α-thalassemia cases. The remaining 10.92% (6/55) of α-carriers had non-deletional mutations, specifically the α-Constant Spring (α-CS) mutation (HBA2: c.427T>C) and the α-WS (α-White Swan, HBA2: c.343_346delCTTC) mutation (Figure 2).

**FIGURE 2:**
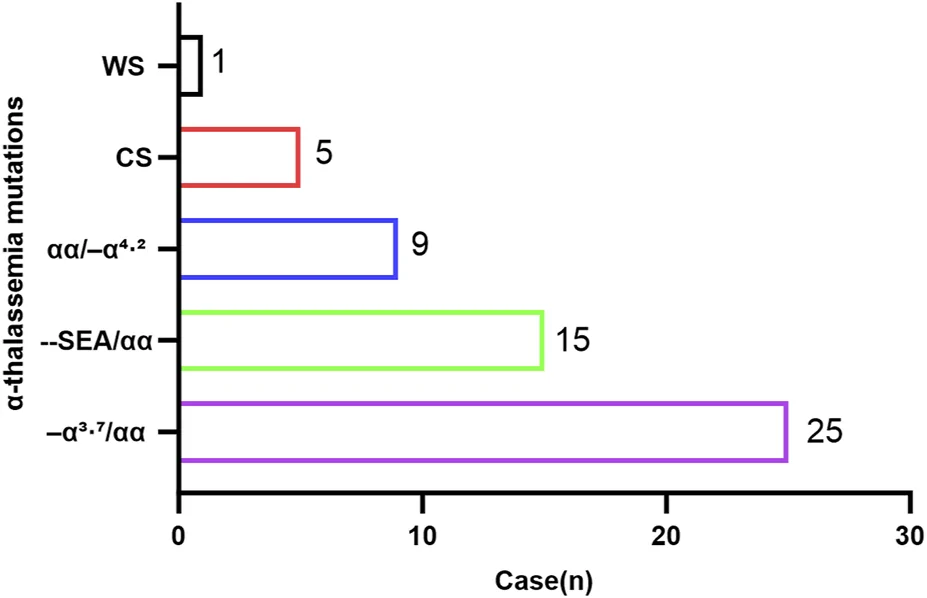
Distribution of α-thalassemia genotypes, Guiyang, China, 2015 (N = 55).

### 3.4 Ethnic disparities in thalassemia carrier rates

Among the 74 identified thalassemia carriers, 34 individuals (45.9%) belonged to ethnic minority groups, representing 10 distinct ethnicities, including Buyi, Miao, Dong, Tujia, and Chuanqing. Carrier rates varied significantly across ethnic groups. The Han population, comprising 685 of the 1,000 neonates (68.5% of the cohort), had a carrier rate of 6.28% (43/685). In contrast, higher carrier rates were observed in several minority groups: Buyi 17.33% (13/75), Dong 26.32% (5/19), Tujia 15.63% (5/32), and Chuanqing 11.54% (3/26). The Miao population had a carrier rate of 7.79% (6/77), similar to that of the Han group (Figure 3). Statistical analysis (chi-square test, SPSS version 23.0) revealed significant differences in carrier rates between the Han, Buyi, Dong, and Tujia populations (overall P < 0.05). Post-hoc pairwise comparisons indicated significantly higher carrier rates in the Buyi, Dong, and Tujia groups compared to the Han population (P < 0.05). No significant difference was found between the Miao and Han populations (P > 0.05) (Table 2).

**FIGURE 3:**
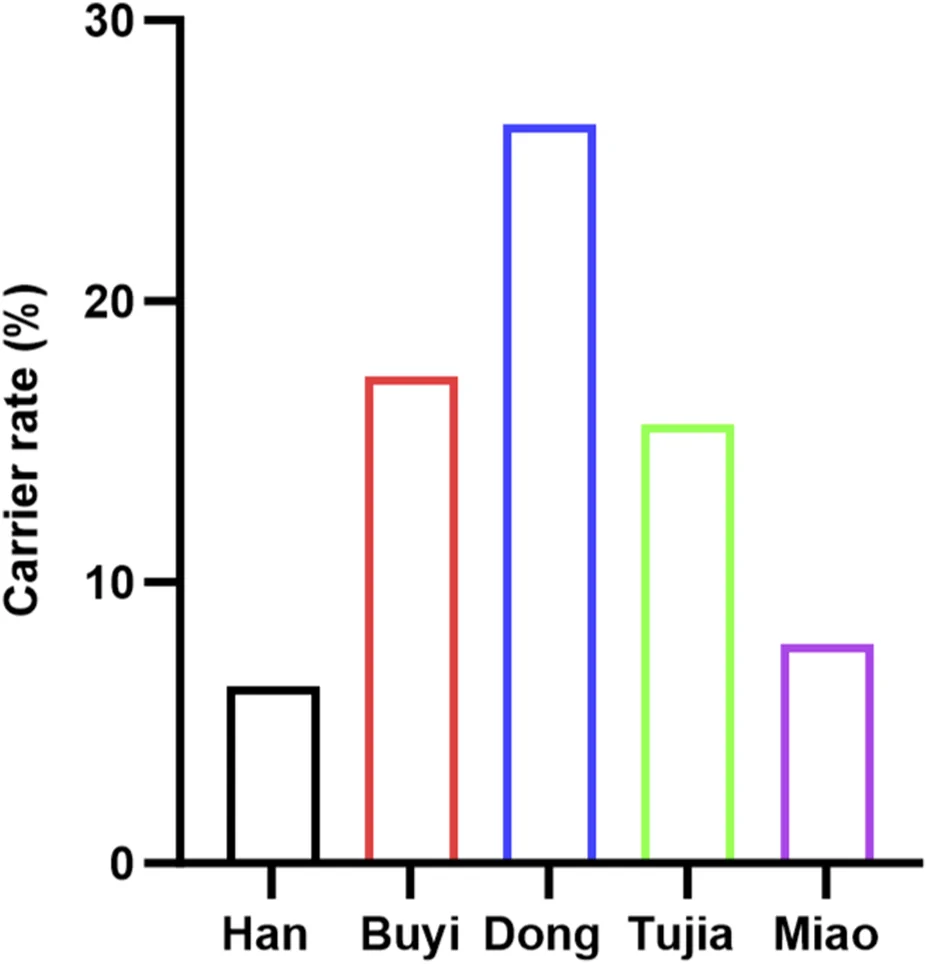
Thalassemia carrier rates stratified by ethnic groups, Guiyang, China, 2015 (N = 1,000).

**TABLE 2:** Comparison of thalassemia carrier rates among different ethnic groups, Guiyang, China, 2015 (N = 1,000).

Ethnic group	Number of subjects (n)	Number of carriers (n)	P value (vs. Han)
Han	685	43	Reference
Buyi	75	13	<0.05
Dong	19	5	<0.01
Tujia	32	5	<0.05
Miao	77	6	>0.05

Overall chi-square test among the five ethnic groups: χ^2^ = 28.36, P < 0.05 (degrees of freedom = 4), indicating a statistically significant difference in carrier rates across groups. Pairwise comparisons were performed using the chi-square test with Bonferroni correction, using the Han group as the reference. Compared with Han, the Buyi, Dong, and Tujia groups showed significantly higher carrier rates (P < 0.05, <0.01, and <0.05, respectively), while the Miao group showed no significant difference (P > 0.05). All statistical analyses were conducted using SPSS 23.0.

### 3.5 MCV-stratified carrier detection: kit versus NGS

All 1,000 cord blood samples were stratified by parental MCV with a cutoff value of 80 fL. A total of 95 neonates had at least one parent with MCV <80 fL. In this subgroup, commercial gene kit detected 19 thalassemia carriers, while NGS identified 29 carriers, with 11 genotypes missed by kit testing and only one variant undetected by NGS. The remaining 905 neonates had parents with normal MCV values. Routine kit screening identified 45 thalassemia carriers in this subgroup, of whom 31 (68.9%) carried silent α-thalassemia variants. Genotype distributions of carriers in both subgroups are presented in Tables 3, 4, respectively.

**TABLE 3:** Comparative thalassemia genotyping results of Kit and NGS in MCV-high-risk neonatal cord blood samples, Guiyang, China, 2015 (N = 95).

Mutation type	Cases detected by kit(n)	Cases detected by NGS(n)	Missed cases by kit(n)
--SEA/αα	9	13	4
–α^3^·^7^/αα	2	3	1
-α^4^·^2^/--SEA	1	1	0
–α^4^·^2^/αα	0	1	1
CD17 (A>T) heterozygous β^0^	5	8	3
CD41-42 (-TTCT) heterozygous β	0	2	2
−28 heterozygous β	1	0	0
Compound heterozygote (--SEA + IVS-II-654)	1	1	0
Total	19	29	11

**TABLE 4:** Thalassemia genotype distribution among neonates with normal parental MCV, Guiyang, China, 2015 (N = 45).

Mutation type	Number of cases (n)
-α^3^·^7^/αα	21
-α^4^·^2^/αα	8
α^CS/αα	5
IVS-II-654 heterozygous β	3
CD17 (A>T) heterozygous β^0^	2
CD41-42 (-TTCT) heterozygous β	2
−28 heterozygous β	1
--SEA/αα	1
α^WS/αα	1
+40–43 indel heterozygous β	1
Total	45

## 4 Discussion

Thalassemia is a hereditary hemolytic anemia with a global distribution, particularly prevalent in Southern China, including Guangdong, Guangxi, Sichuan, Yunnan, and Guizhou. It is classified into α, β, γ, and δ types based on gene deletions, with α and β thalassemia being the most common. Clinically, the disease is categorized into mild, intermediate, and severe forms. Severe cases often result in early death due to chronic hemolysis and recurrent infections. Aside from blood transfusions and allogeneic hematopoietic stem cell transplantation, no radical treatments are available for severe thalassemia. In recent years, large-scale screening programs have been conducted in Guizhou, Guangdong, Guangxi, Hunan, and Chongqing (Mei et al., 2021; Ying et al., 2022; Jiwu et al., 2023; Dan et al., 2024; Yan et al., 2024).

This cord blood-based neonatal epidemiological study identified a regional thalassemia carrier rate of 7.4%, including 5.5% for α-thalassemia, 1.8% for β-thalassemia, and 0.1% for compound αβ-thalassemia. To better interpret these population-based data, we compared our findings with a recent large-scale thalassemia epidemiological study in Guizhou (Zhao et al., 2025). Our neonatal carrier rate (7.4%) was slightly higher than the 6.84% reported in local childbearing-age adults, which is primarily attributed to different study populations and screening approaches; our Kit-NGS strategy detected 11 rare and silent variants missed by conventional commercial kit testing, improving overall detection sensitivity. Despite this minor numerical difference, the two Guizhou cohorts exhibited consistent epidemiological features: α-thalassemia was more prevalent than β-thalassemia, and both studies shared identical predominant variant spectra, with *-α3.7*, *--SEA*, and *-α4.2* as the major α-globin defects, and CD17, CD41-42, and IVS-II-654 as the dominant β-globin mutations. The rare local β-globin variant +40–43 (-AAAC) detected in our neonates was also documented in the adult cohort, confirming its endemic distribution in Guiyang. Both studies revealed significant ethnic disparities in thalassemia prevalence, with higher carrier rates in Dong, Buyi, and Tujia populations than in the Han population, and our neonatal data further demonstrate that such ethnic genetic heterogeneity is congenital rather than acquired. Notably, the two studies complement each other in age coverage: the previous study focused on reproductive-age adults, while our work provides baseline neonatal genetic data that fills the local infant epidemiological gap. Together, they establish a comprehensive cross-age genetic database to support ethnicity-specific precision thalassemia prevention and population screening in Southwest China.

Thalassemia distribution exhibits distinct geographical patterns, influenced by frequent population migration and inter-ethnic marriage in recent years. CD41-42 is the predominant β-thalassemia mutation in Guangdong, Guangxi, Hainan, and Chongqing. The mutation spectrum varies regionally: IVS-II-654, CD17, CD43, and CD71-72 are common in Guangdong (Danqing et al., 2025); CD17, IVS-II-654, and CD71-72 dominate in Guangxi (Dan et al., 2024); CD41-42, IVS-II-654, and CD28 are prevalent in Hainan (Kun et al., 2023). Five major mutations account for over 80% of all β-globin variations in these provinces. Local epidemiological surveys in Guizhou have indicated that CD17 is the most frequent β-thalassemia mutation, followed by CD41-42, IVS-II-654, and HBE (Yan et al., 2024). In line with these findings, our study confirmed CD17 as the leading β-thalassemia genotype, with the three major mutations accounting for 88.9% of all β-thalassemia cases.

Carrier rates of thalassemia vary significantly among ethnic groups. Recent studies have corroborated higher incidence rates in ethnic minority areas of Guizhou compared to other southern regions (Ying et al., 2025). In our study, 34 carriers were from ethnic minorities, with significantly higher carrier rates in the Buyi, Dong, and Tujia populations compared to the Han group (P < 0.05). The observed ethnic heterogeneity in thalassemia carrier prevalence, characterized by significantly higher genetic frequencies among Buyi, Dong, and Tujia populations relative to the Han population, can be explained by multiple long-term historical and population genetic mechanisms. First, these ethnic groups have historically inhabited relatively isolated mountainous regions of southern Guizhou, where geographical enclosure restricted cross-ethnic gene flow with Han populations over generations, resulting in prominent founder effects and progressive enrichment of endemic globin pathogenic variants within local minority genetic pools (Wu et al., 2025). Second, traditional intra-ethnic marriage customs prevalent in remote mountainous settlements historically increased consanguineous mating rates, which facilitated the stable inheritance and accumulation of recessive thalassemia alleles and further elevated carrier frequencies in these subgroups (Kun et al., 2023). Third, the historically high endemicity of malaria in Guizhou’s mountainous areas exerted persistent natural selection pressure; thalassemia heterozygous carriers possess partial resistance against malaria infection, conferring a survival advantage that promoted the retention and enrichment of globin gene variants in local ethnic populations (Zhang et al., 2025). Collectively, geographical isolation, historical consanguineous marriage patterns, and adaptive malaria-driven selection jointly shape the distinct ethnic distribution characteristics of thalassemia variants in the Guizhou region.

In this study, a commercial kit identified 63 carriers, while NGS detected an additional 11 cases, highlighting that reliance solely on the commercial kit may lead to missed diagnoses. The limited mutation coverage and potential technical constraints of the kit likely account for this discrepancy. With its superior sensitivity and broader detection capabilities, NGS offers a valuable complement to conventional methods in neonatal thalassemia screening, reducing the risk of underdiagnosis and facilitating early intervention. We adopted a two-tier screening strategy combining commercial genotyping kits and targeted NGS for 95 neonates with parental MCV <80 fL to optimize thalassemia screening and reduce false-negative results. Prior research has confirmed that MCV-based single hematological screening has prominent defects: silent thalassemia carriers usually show normal red blood cell indices, and iron deficiency interferes with MCV readings, yielding a false-negative rate over 40% (Bharti et al., 2020). In our cohort, only 29 carriers were found among offspring of microcytic parents, while 45 additional carriers were identified from neonates with normal parental MCV, 68.9% of whom carried silent α-thalassemia variants. These data suggest that MCV alone cannot serve as a reliable primary screening marker in thalassemia-endemic areas, and universal genetic testing is recommended for pregnant women and newborns. Standard commercial kits only capture common hotspot variants and fail to detect rare mutations, large copy number variations and complex compound genotypes, which is the leading cause of missed diagnosis (Yang et al., 2025). Targeted NGS covers all globin gene variants to fill this detection gap and improves the identification of rare genotypes, facilitating accurate prenatal thalassemia prevention (Jiang et al., 2023). In the 95 high-risk samples, kits detected 19 carriers versus 29 carriers identified by NGS, with only one variant uniquely captured by the kit, verifying the higher sensitivity of NGS. This MCV-stratified screening workflow balances diagnostic efficiency and economic burden, suitable for grassroots maternal-child care settings.

Further large-scale epidemiological investigations are needed to clarify the exact prevalence in local ethnic minority populations. This study has several inherent limitations that should be acknowledged when interpreting the present epidemiological data. First, all umbilical cord blood samples were recruited from a single urban tertiary maternal and child health hospital in Guiyang, which may introduce single-center selection bias. The enrolled neonatal cohort cannot fully represent the genetic characteristics of newborns residing in rural areas or other prefecture-level cities across Guizhou Province. Second, the routine commercial thalassemia gene kit used for primary screening only contains 23 common pathogenic globin variants prevalent in Chinese populations. Although targeted NGS sequencing was supplemented for high-risk newborns, extremely rare novel globin gene mutations may still escape detection in this workflow. Third, this cross-sectional screening only captured static neonatal carrier genotypes at birth, without long-term longitudinal clinical follow-up of carriers and their offspring. Therefore, we cannot evaluate the long-term phenotypic progression and reproductive risks of these thalassemia carriers. Fourth, the sample sizes of several ethnic minority subgroups were relatively unbalanced. Small participant numbers in some minor ethnic groups may reduce the statistical power of intergroup comparisons of thalassemia carrier rates. Last, the sample size of the high-risk subgroup was limited, and silent carriers with normal parental MCV were not separately analyzed. Further large-scale multicenter studies incorporating hemoglobin electrophoresis and serum ferritin will refine screening protocols and lower overall false-negative rates. Considering these limitations, future multi-center population-based screening studies covering both urban and rural regions of Guizhou are required to obtain more comprehensive and generalizable baseline data of thalassemia mutation spectrum among multi-ethnic newborns.

This study provides valuable insights into the thalassemia genetic background at the neonatal level, enriching local genetic data resources. It also lays the foundation for genetic counseling, follow-up treatment, and further epidemiological research in the region.

## 5 Conclusion

This cross-sectional study establishes the comprehensive baseline of neonatal thalassemia carrier status in Guiyang, delineating a distinct mutational landscape characterized by heterogeneous α/β-globin gene variants and significant inter-ethnic disparities. Notably, the integrated two-tier screening algorithm—combining kit and NGS—substantially enhances the detection of rare and silent alleles, underscoring its clinical utility over conventional approaches. Collectively, our findings advocate for a tiered prevention framework tailored to Guizhou’s multi-ethnic demographic: universal neonatal molecular screening, reflexive NGS for high-risk infants, and ethnicity-sensitive preconception genetic counseling. Implementation of this evidence-based strategy holds promise for effectively curbing the incidence of severe thalassemia and alleviating the long-term disease burden in this underserved mountainous region.

## Data Availability

The original contributions presented in the study are included in the article/supplementary material, further inquiries can be directed to the corresponding author.
